# Elastic anisotropy in the reduced Landau–de Gennes model

**DOI:** 10.1098/rspa.2021.0966

**Published:** 2022-05

**Authors:** Yucen Han, Joseph Harris, Apala Majumdar, Lei Zhang

**Affiliations:** ^1^ Department of Mathematics and Statistics, University of Strathclyde, Glasgow G1 1XQ, UK; ^2^ Beijing International Center for Mathematical Research, Center for Quantitative Biology, Peking University, Beijing 100871, People’s Republic of China

**Keywords:** nematic liquid crystal, Landau–de Gennes, elastic anisotropy, asymptotic analyses, bifurcation diagrams

## Abstract

We study the effects of elastic anisotropy on Landau–de Gennes critical points, for nematic liquid crystals, on a square domain. The elastic anisotropy is captured by a parameter, $L_{2}$, and the critical points are described by 3 d.f. We analytically construct a symmetric critical point for all admissible values of $L_{2}$, which is necessarily globally stable for small domains, i.e. when the square edge length, λ, is small enough. We perform asymptotic analyses and numerical studies to discover at least five classes of these symmetric critical points—the WORS, ${\text{Ring}}^{\pm }$, Constant and pWORS solutions, of which the WORS, ${\text{Ring}}^{+}$ and Constant solutions can be stable. Furthermore, we demonstrate that the novel Constant solution is energetically preferable for large λ and large $L_{2}$, and prove associated stability results that corroborate the stabilizing effects of $L_{2}$ for reduced Landau–de Gennes critical points. We complement our analysis with numerically computed bifurcation diagrams for different values of $L_{2}$, which illustrate the interplay of elastic anisotropy and geometry for nematic solution landscapes, at low temperatures.

## 1. Introduction

Nematic liquid crystals (NLCs) are quintessential examples of partially ordered materials that combine fluidity with the directionality of solids [1]. The nematic molecules are typically asymmetric in shape, e.g. rod- or disc-shaped, and these molecules tend to align along certain locally preferred averaged directions, referred to as *nematic directors* in the literature. Consequently, NLCs have a long-range orientational order and direction-dependent physical, optical and rheological properties. It is precisely this anisotropy that makes them the working material of choice for a range of electro-optic devices such as the multi-billion dollar liquid crystal display industry [2,3].

There has been substantial recent interest in multistable NLC systems, i.e. NLCs, confined to two-dimensional (2D) or three-dimensional (3D) geometries that can support multiple stable states without any external electric fields [4–10]. Multistable NLC systems offer new prospects for device technologies, materials technologies, self-assembly processes and hydrodynamics. This paper is motivated by a bistable system reported in [11]. Here, the authors experimentally and numerically study NLCs inside periodic arrays of 3D wells, with a square cross-section, such that the well height is typically much smaller than the square cross-sectional length. Furthermore, the authors speculate that the structural characteristics are translationally invariant along the well-height, effectively reducing this to a 2D problem. Hence, the authors restrict attention to the bottom square cross-section with square edge length denoted by λ, which is typically on the micron scale. The choice of boundary conditions is crucial and in [11], the authors impose tangent boundary conditions (TBCs) on the well surfaces, i.e. the nematic directors, in the plane of the well surfaces, are constrained to be in the plane of the surfaces. However, this necessarily means that the nematic director is tangent to the square edges, creating defects at the vertices, where the director is not defined. The authors observe two classes of stable NLC states: the diagonal D states, for which the nematic director aligns along one of the square diagonals and; the rotated R states, for which the director rotates by π radians between a pair of opposite square edges.

In [6,12], the authors model this square system within the celebrated continuum Landau–de Gennes (LdG) theory for NLCs. The LdG theory describes the nematic state by a macroscopic order parameter—the Q-tensor-order parameter [1]. From an experimental perspective, the Q-tensor is measured in terms of NLC responses to external electric or magnetic fields, which are necessarily anisotropic in nature. Mathematically, the Q-tensor-order parameter is a symmetric, traceless, 3×3 matrix with 5 d.f. For a square domain with TBCs on the square edges, it suffices to work in a reduced LdG framework where the Q-tensor only has three degrees of freedom, $q_{1}$, $q_{2}$, $q_{3}$. The degree of nematic order in the plane is captured by $q_{1}$ and $q_{2}$, whereas $q_{3}$ measures the out-of-plane order, such that positive (negative) $q_{3}$ implies that the nematic director lies out of the plane (in the plane) of the square, respectively. The TBCs naturally constrain $q_{3}$ to be negative on the square edges, but $q_{3}$ could be positive in the interior.

The LdG theory is a variational theory, i.e. experimentally observable states can be modelled by local or global minimizers of an appropriately defined LdG free energy. In the simplest setting, the LdG energy has two contributions—a bulk energy and an elastic energy that penalizes spatial inhomogeneities. In these papers, the authors work with low temperatures that favour an ordered nematic state. The elastic energy is typically a quadratic and convex function of ∇Q and, in [6,12], the authors work with an isotropic elastic energy—the Dirichlet elastic energy. In a reduced LdG setting, the authors recover the stable D and R states for large λ and, in [12], they discover a novel stable Well Order Reconstruction Solution (WORS) for small λ. The WORS is special because it exhibits a pair of mutually orthogonal defect lines, with no planar nematic order along the defect lines as will be described in §3. In [13], the authors generalize this work to arbitrary 2D regular polygons and, in [8], the authors study 3D wells, with an emphasis on novel mixed solutions which interpolate between two distinct D solutions.

In this paper, we study the same problem of NLCs on a square domain with TBCs, with an anisotropic elastic energy as opposed to the isotropic energy studied in [6,12]. Notably, we take the elastic energy density to be $w(\nabla Q)=|\nabla Q{|}^{2}+L_{2}{\left(\text{div}Q\right)}^{2}$, where $L_{2}>-1$ is the anisotropy parameter. Physically speaking, positive $L_{2}$ implies that splay and bend deformations of the nematic director are energetically expensive compared to out-of-plane twist deformations, i.e. we expect the physically observable states to have positive $q_{3}$ in the square interior, as $L_{2}$ increases. Therefore, there are competing effects of the TBCs on the square edges, which prefer in-plane director orientation, and the preferred out-of-plane director orientation in the square interior, for larger values of $L_{2}$. We construct a symmetric critical point of the LdG energy, for any $L_{2}>-1$. This symmetric critical point is globally stable for λ small enough. The WORS is a special case of this symmetric critical point with $q_{2}\equiv 0$ on the square domain, for $L_{2}=0$. For $L_{2}\neq 0$, the *WORS* does not survive with the perfect cross symmetry along the square diagonals. We perform an asymptotic analysis for small λ and small $L_{2}$, about the WORS. The anisotropy has a first-order effect on $q_{3}$, i.e. $q_{3}$ is perturbed linearly by $L_{2}$, and $q_{3}$ increases at the square centre for positive $L_{2}$, relative to its value for $L_{2}=0$, corroborating the trend of increasing $q_{3}$ with increasing $L_{2}$. The globally stable symmetric critical point for small λ and small $L_{2}\neq 0$, labelled as the $Ring^{+}$ solution, effectively smoothens out the WORS and exhibits a stable central +1-degree point defect. We perform formal calculations to show that as $L_{2}\to \infty$, energy minimizers (and consequently the symmetric critical point described above for small λ) approach the Constant state with $\left(q_{1},q_{2},q_{3})=(0,0,s_{+}/3\right)$, away from the square edges and exhibits four boundary layers near the edges. Thus, there are three different classes of the symmetric critical point discussed above: the WORS, which only exists for $L_{2}=0$; the $Ring^{+}$ solution, which only can be stable for moderate values of λ and non-zero $L_{2}$ and; the Constant solution, which exists for $L_{2}$ large enough and is always stable according to our heuristics and numerical calculations. Additionally, we also find two unstable classes of this symmetric critical point, both of which exist for moderate values of λ and $L_{2}$. These are the $Ring^{-}$ solution which exhibits a central −1-degree point defect, and the novel pWORS which exhibits an oscillating sequence of nematic point defects along the square diagonals. We provide asymptotic approximations for the novel pWORS solution branch.

While most of our work is restricted to the small λ-limit, we also touch on energy minimizers in the λ→∞ limit. The competitors in the large λ-limit are the familiar D and R states, and the Constant solution. Using Gamma-convergence arguments, we show that the Constant solution has lower energy than the D and R states, for large enough $L_{2}$. We complement our analysis with numerical computations of bifurcation diagrams for five different values of $L_{2}$. To summarize, our notable findings on the response of the NLC solution landscape for this model problem, to the elastic anisotropy parameter, $L_{2}$, are (i) novel stable states (${\text{Ring}}^{+}$ and Constant) for small λ, and (ii) enhanced multistability in the large λ-limit due to the competing Constant and ${\text{Ring}}^{+}$ states, for large $L_{2}$. As $L_{2}$ increases, we expect that there are further, not necessarily energy-minimizing, LdG critical points with positive $q_{3}$, or out-of-plane nematic directors in the square interior. Furthermore, $L_{2}$ has a stabilizing effect with respect to certain classes of planar perturbations and out-of-plane perturbations, and so we expect enhanced multistability as $L_{2}$ increases, for all values of λ.

A lot of open questions remain with regards to the interplay between $L_{2}$, λ and temperature on NLC solution landscapes, but our work is an informative forward step in this direction. Our paper is organized as follows. We provide all the mathematical preliminaries in §2. We construct the symmetric critical points described above in §3. In §4, we perform separate asymptotic studies in the small λ and small $L_{2}$ limit; large $L_{2}$ limit; large λ-limit. In §5, we present bifurcation diagrams for five different values of $L_{2}$, accompanied by some rigorous stability results. We conclude with some perspectives in §6.

## 2. Preliminaries

In this section, we review the LdG theory of NLCs. Within this framework, the nematic state is described by a macroscopic LdG order parameter—the Q-tensor-order parameter. The Q-tensor is a symmetric, traceless, 3×3 matrix, which is a macroscopic measure of the nematic anisotropy. The eigenvectors of Q represent the preferred material directions, the corresponding eigenvalues measure the degree of order about these directions. The nematic director is often identified with the eigenvector that has the largest positive eigenvalue. The Q-tensor is said to be: (i) isotropic if Q=0; (ii) uniaxial if Q has a pair of degenerate non-zero eigenvalues; and (iii) biaxial if Q has three distinct eigenvalues. A uniaxial Q-tensor can be written as $Q_{u}=s(n⊗n-I/3)$, where I is the 3×3 identity matrix, $n\in S^{2}$ is the distinguished eigenvector with the non-degenerate eigenvalue, and s∈R is a scalar order parameter. The LdG theory is a variational theory with an associated free energy, and the basic modelling hypothesis is that the physically observable configurations correspond to global or local energy minimizers subject to imposed boundary conditions. We work with 2D domains, $\Omega \subset R^{2}$, in the context of modelling *thin* 3D systems. In the absence of a surface anchoring energy and external fields, the LdG free energy is given by

2.1 $$F[\text{Q}]:=\int_{\Omega }f_{\mathrm{el}}(\text{Q},\nabla \text{Q})+f_{b}(\text{Q})\;\mathrm{dA},$$

where $f_{\mathrm{el}}$ and $f_{b}$ are the elastic and thermotropic bulk energy densities, respectively. We consider a two-term elastic energy density:

2.2 $$f_{\mathrm{el}}(\text{Q})=\frac{L}{2}(|\nabla \text{Q}{|}^{2}+L_{2}{\left(\mathrm{div}\text{Q}\right)}^{2}),$$

where L>0 is an elastic constant, and $L_{2}\in (-1,\infty )$ is the ‘elastic anisotropy’ parameter. The elastic energy density penalizes spatial inhomogeneities, typically quadratic in ∇Q. In terms of notation, we use $|\nabla Q{|}^{2}:=(\partial Q_{ij}/\partial x_{k})(\partial Q_{ij}/\partial x_{k})$ and ${\left(\mathrm{div}Q\right)}^{2}:=(\partial Q_{ij}/\partial x_{j})(\partial Q_{ik}/\partial x_{k})$, i,j,k=1,2,3, where the Einstein summation convention is assumed throughout this manuscript. We work with a 2D confining geometry Ω in this paper and hence, $\partial Q_{ij}/\partial x_{3}=0$ for all 1≤i,j≤3. We work with the simplest form of $f_{b}$, that allows for a first-order isotropic–nematic transition:

2.3 $$f_{b}(\text{Q}):=\frac{A}{2}\mathrm{tr}{\text{Q}}^{2}-\frac{B}{3}\mathrm{tr}{\text{Q}}^{3}+\frac{C}{4}{\left(\mathrm{tr}{\text{Q}}^{2}\right)}^{2}.$$

Here, $\mathrm{tr}Q^{2}=Q_{ij}Q_{ij}$, and $\mathrm{tr}Q^{3}=Q_{ij}Q_{jk}Q_{ki}$, for i,j,k=1,2,3. We take $A=\alpha (T-T^{*})$ to be the rescaled temperature and α,B,C>0 are material-dependent constants. In this regime, T is the absolute temperature, and $T^{*}$ is the characteristic nematic supercooling temperature. The rescaled temperature, A, has three physically relevant values: (i) A=0, below which the isotropic state, Q=0, loses stability; (ii) the nematic super-heating temperature $A=B^{2}/24C$, above which Q=0 is the unique critical point of $f_{b}$ and (iii) the nematic-isotropic phase transition temperature $A=B^{2}/27C$, at which $f_{b}$ is minimized by the isotropic phase and a continuum of uniaxial states. We work with low temperatures, A<0, for which $f_{b}$ is minimized on the set of uniaxial Q-tensors defined by $N:=\{Q\in S_{0}:Q=s_{+}(n⊗n-I/3)\}$ where $S_{0}$ is the space of traceless symmetric 3×3 matrices and

2.4 $$s_{+}=\frac{B+\sqrt{B^{2}+24|A|C}}{4C},\;\text{n}\in S^{2}\;\text{arbitrary}.$$

We non-dimensionalize the system using, $\bar{x}=x/\lambda$, where λ is a characteristic geometrical length-scale, e.g. half edge length of a 2D regular polygon. The rescaled LdG energy functional (up to a multiplicative constant) is given by

2.5 $$F_{\lambda }[\text{Q}]:=\int_{\bar{\Omega }}\left\{\frac{1}{2}|\nabla_{\bar{x}}\text{Q}{|}^{2}+\frac{L_{2}}{2}{\left({\mathrm{div}}_{\bar{x}}\text{Q}\right)}^{2}+\frac{\lambda^{2}}{L}f_{b}(\text{Q})\right\}\;d\bar{A},$$

where $\bar{\Omega }$ is the rescaled domain in $R^{2}$, and $d\bar{A}$ is the rescaled area element. We drop the ‘bars’ but all computations should be interpreted in terms of the rescaled variables.

Next, we define the working domain and Dirichlet boundary conditions, although we believe that our methods can be generalized to arbitrary 2D domains. We focus on square domains, building on the substantial work in [7,14,15]. We impose almost uniaxial Dirichlet TBCs on the square edges, which require the nematic director to be tangent to the edges, necessarily creating a mismatch at the square vertices. This is consistent with the experimentally and numerically investigated TBCs, [6,11,12]. To avoid the discontinuities at the vertices, we take $\Omega \subset R^{2}$ to be a truncated square whose edges are parallel to the coordinate axes:

2.6 $$\Omega :=\{(x,y)\in R^{2}:|x|<1,|y|<1,|x+y|<2-\varepsilon ,|x-y|<2-\varepsilon \}.$$

**Figure 1.**
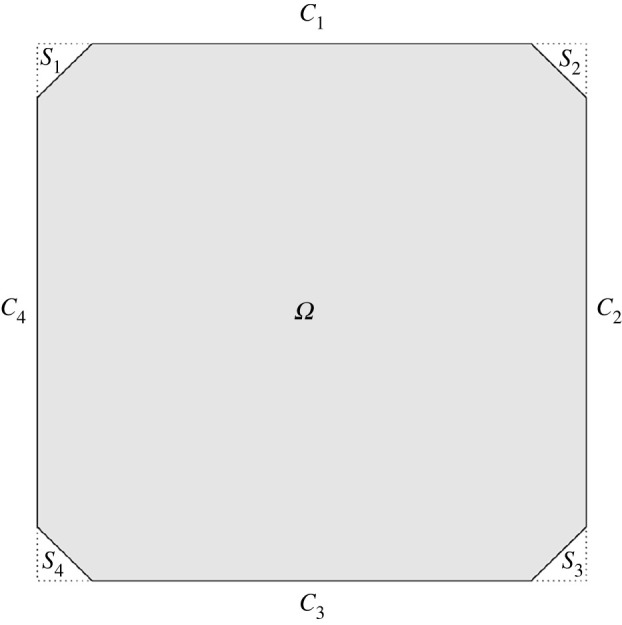
The truncated square domain Ω.

Provided ε≪1, the truncation does not change the qualitative properties of the LdG energy minimizers away from the square vertices. The boundary, ∂Ω, has four ‘long’ edges parallel to the coordinate axes, defined in a clockwise fashion as $C_{1},\ldots ,C_{4}$, where $C_{1}$ lies parallel to the x-axis at y=1. The truncation creates four additional ‘short’ edges, of length $\sqrt{2}\varepsilon$, parallel to the lines y=x and y=−x, labelled as $S_{1},\ldots ,S_{4}$ in a clockwise fashion, starting at the top-left corner of the domain. The domain is illustrated in figure 1. In particular, we fix the uniaxial director to be n=(±1,0) on the edges, $C_{1}$ and $C_{3}$, and n=(0,±1) on $C_{2}$ and $C_{4}$. From a physical standpoint, this constitutes *strong* (infinite) anchoring on the long edges. One could also model weak (finite) anchoring condition with an additional surface energy in the LdG free energy [16], but that would make the analysis more complicated. We set

2.7 $$\text{Q}={\text{Q}}_{b}\;\text{on}\;\partial \Omega ,$$

where

2.8 $${\text{Q}}_{b}(x,y):=\left\{ \begin{array}{ll} s_{+}\left(\hat{\text{x}}⊗\hat{\text{x}}-\text{I}/3\right), & (x,y)\in C_{1}\cup C_{3},\\ s_{+}\left(\hat{\text{y}}⊗\hat{\text{y}}-\text{I}/3\right), & (x,y)\in C_{2}\cup C_{4}, \end{array}\right.$$

where $\hat{x}$ and $\hat{y}$ are unit vectors in the x- and y-directions, respectively. In particular, $Q_{b}\in N$ on $C_{1},\ldots ,C_{4}$. On the short edges, $S_{1},\ldots ,S_{4}$, we prescribe a continuous interpolation between the boundary conditions on the long edges (2.8) given by

2.9 $${\text{Q}}_{b}(x,y):=\left\{ \begin{array}{ll} g(x+y)(\hat{\text{x}}⊗\hat{\text{x}}-\hat{\text{y}}⊗\hat{\text{y}})-\frac{s_{+}}{2}\left(\hat{\text{z}}⊗\hat{\text{z}}-\frac{\text{I}}{3}\right), & (x,y)\in S_{1},\\ g(y-x)(\hat{\text{x}}⊗\hat{\text{x}}-\hat{\text{y}}⊗\hat{\text{y}})-\frac{s_{+}}{2}\left(\hat{\text{z}}⊗\hat{\text{z}}-\frac{\text{I}}{3}\right), & (x,y)\in S_{2},\\ g(-x-y)(\hat{\text{x}}⊗\hat{\text{x}}-\hat{\text{y}}⊗\hat{\text{y}})-\frac{s_{+}}{2}\left(\hat{\text{z}}⊗\hat{\text{z}}-\frac{\text{I}}{3}\right), & (x,y)\in S_{3},\\ g(x-y)(\hat{\text{x}}⊗\hat{\text{x}}-\hat{\text{y}}⊗\hat{\text{y}})-\frac{s_{+}}{2}\left(\hat{\text{z}}⊗\hat{\text{z}}-\frac{\text{I}}{3}\right), & (x,y)\in S_{4}, \end{array}\right.$$

where $\hat{z}$ is a unit vector in the z-direction, and $g:[-\varepsilon ,\varepsilon ]\to [-s_{+}/2,s_{+}/2]$ is a smoothing function, i.e. $g(l)=(s_{+}/2\varepsilon )l,-\varepsilon \leq l\leq \varepsilon .$ Although the boundary conditions (2.9) do not minimize $f_{b}$ on $S_{1},\ldots ,S_{4}$, and do not respect TBCs, they are short by construction and are chosen purely for mathematical convenience. Given the Dirichlet boundary conditions (2.8) and (2.9), the admissible space is

2.10 $$A:=\{\text{Q}\in W^{1,2}(\Omega ;S_{0}):\text{Q}={\text{Q}}_{b}\;\text{on}\;\partial \Omega \}.$$

The energy minimizers, or indeed any critical point of the LdG energy (2.5), are solutions of the associated Euler–Lagrange equations:

2.11 $$\begin{array}{r} \Delta Q_{ij}+\frac{L_{2}}{2}\left(Q_{ik,kj}+Q_{jk,ki}-\frac{2}{3}\delta_{ij}Q_{kl,kl}\right)=\frac{\lambda^{2}}{L}\left\{AQ_{ij}-B\left(Q_{ik}Q_{kj}-\frac{1}{3}\delta_{ij}\mathrm{tr}{\text{Q}}^{2}\right)+CQ_{ij}\mathrm{tr}{\text{Q}}^{2}\right\}, \end{array}$$

which comprise a system of five nonlinear coupled partial differential equations. The terms $(2/3)Q_{kl,kl}$ and $(1/3)\mathrm{tr}Q^{2}$ are Lagrange multipliers associated with the tracelessness constraint.

Finally, we comment on the physical relevance of the 2D domain, $\Omega \subset R^{2}$. Consider a 3D well,

$$B=\{(x,y,z)\in R^{3};(x,y)\in \Omega ;z\in (0,h)\},$$

where h≪λ, and λ is a characteristic length scale of Ω. In this limit, one can assume (at least for modelling purposes) that physically relevant Q-tensors are independent of the z-coordinate, i.e. the profiles are invariant across the height of the well, and that $\hat{z}$ is a fixed eigenvector (see [17,18] for some rigorous analysis and justification). This implies that we can restrict ourselves to Q-tensors with three degrees of freedom:

2.12 $$\begin{array}{rl} & \text{Q}=q_{1}(x,y)(\hat{\text{x}}⊗\hat{\text{x}}-\hat{\text{y}}⊗\hat{\text{y}})+q_{2}(x,y)(\hat{\text{x}}⊗\hat{\text{y}}+\hat{\text{y}}⊗\hat{\text{x}})+q_{3}(x,y)(2\hat{\text{z}}⊗\hat{\text{z}}-\hat{\text{x}}⊗\hat{\text{x}}-\hat{\text{y}}⊗\hat{\text{y}}), \end{array}$$

subject to the boundary conditions

2.13 $$q_{1}(x,y)=q_{b}(x,y),\;\text{on}\;\partial \Omega$$

where $q_{b}=s_{+}/2$, on $C_{1}\cup C_{3}$; $q_{b}=-s_{+}/2$, on $C_{2}\cup C_{4}$; $q_{b}=g(x+y)$, on $S_{1}$; $q_{b}=g(y-x)$, on $S_{2}$; $q_{b}=g(-x-y)$, on $S_{3}$; $q_{b}=g(x-y)$, on $S_{4}$, and

2.14 $$q_{2}=0,\;\text{and}\;q_{3}(x,y)=\frac{-s_{+}}{6}\;\text{on}\;\partial \Omega .$$

The conditions (2.13) and (2.14) are equivalent to Dirichlet conditions in (2.7).

## 3. Qualitative properties of equilibrium configurations

In [12], the authors numerically compute critical points of (2.5) with $L_{2}=0$, satisfying the Dirichlet boundary conditions (2.7), on the square cross-section Ω. For the edge length, λ small enough, the authors report a new WORS. The WORS has a constant set of eigenvectors, $\hat{x},\hat{y}$ and $\hat{z}$, which are the coordinate unit vectors. The WORS is further distinguished by a uniaxial cross, with negative scalar order parameter, along the square diagonals. Physically, this implies that there is a planar defect cross along the square diagonals, and the nematic molecules are disordered along the square diagonals. In [7], the authors analyse this system at a fixed temperature $A=-B^{2}/3C$ with $L_{2}=0$, and show that the WORS is a classical solution of the associated Euler–Lagrange equations (2.11) of the form:

3.1 $${\text{Q}}_{\text{WORS}}(x,y)=q(\hat{\text{x}}⊗\hat{\text{x}}-\hat{\text{y}}⊗\hat{\text{y}})-\frac{B}{6C}(2\hat{z}⊗\hat{z}-\hat{\text{x}}⊗\hat{\text{x}}-\hat{\text{y}}⊗\hat{\text{y}}).$$

The single degree of freedom, q:Ω→R, is a solution of the Allen–Cahn equation with the following symmetry properties:

3.2 $$q=0\;\text{on}\;\{y=x\}\cup \{y=-x\},\;(y^{2}-x^{2})q(x,y)\geq 0.$$

Notably, $q_{2}=0$ everywhere for the WORS (refer to (2.12)), which is equivalent to having a set of constant eigenvectors. They prove that the WORS is globally stable for λ small enough, and unstable for λ large enough, demonstrating a pitchfork bifurcation in a scalar setting. Their analysis is restricted to the specific temperature and, in [8], the authors extend the analysis to all A<0, with $L_{2}=0$. In this section, we analyse the equilibrium configurations with $L_{2}\neq 0$, including their symmetry properties in the small λ limit. Notably, we show that the cross structure of the WORS does not survive with $L_{2}\neq 0$.

**Proposition 3.1.**

*There exists at least one solution to the Euler–Lagrange equations* (*2.11*) *of the form* (*2.12*) *in* A, *given the Dirichlet boundary conditions* (*2.8*) *and* (*2.9*). *For this solution, the functions* $q_{1},q_{2},q_{3}$ *satisfy the following systems of PDEs*:

3.3 $$\begin{array}{rl} & \left(1+\frac{L_{2}}{2}\right)\Delta q_{1}+\frac{L_{2}}{2}(q_{3,yy}-q_{3,xx})=\frac{\lambda^{2}}{L}q_{1}(A+2Bq_{3}+2C(q_{1}^{2}+q_{2}^{2}+3q_{3}^{2})), \end{array}$$

3.4 $$\begin{array}{rl} & \left(1+\frac{L_{2}}{2}\right)\Delta q_{2}-L_{2}q_{3,xy}=\frac{\lambda^{2}}{L}q_{2}(A+2Bq_{3}+2C(q_{1}^{2}+q_{2}^{2}+3q_{3}^{2})) \end{array}$$

3.5 $$\begin{array}{rl} & \left(1+\frac{L_{2}}{6}\right)\Delta q_{3}+\frac{L_{2}}{6}(q_{1,yy}-q_{1,xx})-\frac{L_{2}}{3}q_{2,xy}=\frac{\lambda^{2}}{L}q_{3}(A-Bq_{3}+2C(q_{1}^{2}+q_{2}^{2}+3q_{3}^{2}))\\ & \;\;+\frac{\lambda^{2}B}{3L}(q_{1}^{2}+q_{2}^{2}), \end{array}$$

*and the boundary conditions* (2.13) *and* (2.14).

**Proof.**

Our proof is analogous to theorem 2.2 in [18]. Substituting the Q-tensor ansatz (2.12) into the general form of the LdG energy (2.5), let

3.6 $$J[q_{1},q_{2},q_{3}]:=\int_{\Omega }f_{\mathrm{el}}(q_{1},q_{2},q_{3})+\frac{\lambda^{2}}{L}f_{b}(q_{1},q_{2},q_{3})\;dA,$$

where

3.7 $$f_{\mathrm{el}}(q_{1},q_{2},q_{3}):=|\nabla q_{1}{|}^{2}+|\nabla q_{2}{|}^{2}+3|\nabla q_{3}{|}^{2}+\frac{L_{2}}{2}({\left(q_{1,x}+q_{2,y}-q_{3,x}\right)}^{2}+{\left(q_{2,x}-q_{1,y}-q_{3,y}\right)}^{2}),$$

and

3.8 $$f_{b}(q_{1},q_{2},q_{3}):=A(q_{1}^{2}+q_{2}^{2}+3q_{3}^{2})+C{\left(q_{1}^{2}+q_{2}^{2}+3q_{3}^{2}\right)}^{2}+2Bq_{3}(q_{1}^{2}+q_{2}^{2}-q_{3}^{2}),$$

are the elastic and thermotropic bulk energy densities, respectively. We prove the existence of minimizers of J in the admissible class

3.9 $$A_{0}:=\{(q_{1},q_{2},q_{3})\in W^{1,2}(\Omega ;R^{3}):q_{1}=q_{b},\;q_{2}=0,\;q_{3}=\frac{-s_{+}}{6}\;\text{on}\;\partial \Omega \},$$

which will also be solutions of (2.11) in the admissible space, A. Since the boundary conditions (2.13) and (2.14) are piece-wise of class $C^{1}$, $A_{0}$ is non-empty. If $L_{2}\in [0,\infty )$, $f_{\mathrm{el}}$ is in the form of (3.7). If $L_{2}\in (-1,0)$, the elastic energy density can be rewritten as a function of $\left(q_{1},q_{2},q_{3})\in W^{1,2}(\Omega ;R^{3}\right)$ in the following way:

3.10 $$\begin{array}{rl} f_{\mathrm{el}} & \;=(1+L_{2})(|\nabla q_{1}{|}^{2}+|\nabla q_{2}{|}^{2}+3|\nabla q_{3}{|}^{2})\\ & \;\;-\frac{L_{2}}{2}{\left((-q_{3,x}-q_{1,x}-q_{2,y}\right)}^{2}+{\left(q_{2,x}-q_{1,y}+q_{3,y}\right)}^{2}+4|\nabla q_{3}{|}^{2}). \end{array}$$

The difference between the expressions for $f_{\mathrm{el}}$ in (3.7) and (3.10), is a null Lagrangian, and hence can be ignored with the Dirichlet boundary condition. Since we assume that $1+L_{2}>0$, the elastic energy density is the sum of non-negative terms for any $L_{2}>-1$ and, more specifically,

3.11 $$f_{\mathrm{el}}(q_{1},q_{2},q_{3})\geq \min\{1,1+L_{2}\}(|\nabla q_{1}{|}^{2}+|\nabla q_{2}{|}^{2}+3|\nabla q_{3}{|}^{2}).$$

Furthermore, $f_{b}$ also satisfies $f_{b}(q_{1},q_{2},q_{3})\geq f_{b}(\pm (s_{+}/2),0,-(s_{+}/6))=:M_{1}(A,B,C),$ for some constant, $M_{1}>0$, depending only on A,B and C. Hence $J[q_{1},q_{2},q_{3}]$ is coercive in $A_{0}$. Finally, we note that J is weakly lower semi-continuous on $W^{1,2}(\Omega )$, which follows immediately from the fact that $f_{\mathrm{el}}$ is quadratic and convex in $\nabla (q_{1},q_{2},q_{3})$. Thus, the direct method in the calculus of variations yields the existence of a global minimizer of J, among the finite energy triplets $\left(q_{1},q_{2},q_{3})\in W^{1,2}(\Omega ;R^{3}\right)$, satisfying the boundary conditions (2.13) and (2.14) [19]. One can verify that the semilinear elliptic system (3.3)–(3.5) are the Euler–Lagrange equations associated with J, and the minimizers for J are $C^{\infty }(\Omega )\cap C^{2}(\bar{\Omega })$ solutions of (3.3)–(3.5). The corresponding Q-tensor (2.12) is an exact solution of the LdG Euler–Lagrange equations (2.11).

**Proposition 3.2.**

*There exists a critical point* $\left(q_{1}^{s},q_{2}^{s},q_{3}^{s}\right)$ *of the energy functional* (3.6) *in* $A_{0}$, *for all* λ>0, *such that* $q_{1}=0$ *on the square diagonals* y=x *and* y=−x, *and* $q_{2}=0$ *on* x=0 *and* y=0.

**Proof.**

We follow the approach in [7]. Consider 1/8th of a square located in the positive quadrant of Ω:

3.12 $$\Omega_{q}:=\{(x,y)\in \Omega :0<y<x,\;0<x<1\}.$$

The following boundary conditions on $\Omega_{q}$ are consistent with the boundary conditions (2.13) and (2.14) on the whole of Ω:

3.13 $$\begin{array}{rl} & q_{1}=q_{b},\;q_{2}=0,\;q_{3}=-\frac{s_{+}}{6},\;(x,y)\in \partial \Omega_{q}\cap \partial \Omega ;\\ & q_{1}=\partial_{\nu }q_{2}=\partial_{\nu }q_{3}=0,\;\;(x,y)\in \partial \Omega_{q}\cap \{y=x\}\\ \mathrm{and}\; & \partial_{\nu }q_{1}=q_{2}=\partial_{\nu }q_{3}=0,\;\;(x,y)\in \partial \Omega_{q}\cap \{y=0\}, \end{array}\}$$

where $\partial_{\nu }$ represents the outward normal derivative. We minimize the associated LdG energy in $\Omega_{q}$, given by

3.14 $$J[q_{1},q_{2},q_{3}]=\int_{\Omega_{q}}f_{\mathrm{el}}(q_{1},q_{2},q_{3})+\frac{\lambda^{2}}{L}f_{b}(q_{1},q_{2},q_{3})\;\mathrm{dA},$$

on the admissible space $A_{q}:=\{(q_{1},q_{2},q_{3})\in W^{1,2}(\Omega_{q};R^{3}):\text{( 3.13) }\;\text{is satisfied}\}.$ As the boundary conditions on $\Omega_{q}$ are continuous and piecewise of class $C^{1}$, $A_{q}$ is non-empty. Furthermore, we have shown that J is coercive on $A_{q}$ and convex in the gradient $\nabla (q_{1},q_{2},q_{3})$. Thus, by the direct method in the calculus of variations, there exists a minimizer $(q_{1}^{*},q_{2}^{*},q_{3}^{*})\in A_{q}$. We define a function $q_{1}^{s}\in \Omega$ by odd reflection of $q_{1}^{*}\in \Omega_{q}$ about the square diagonals, and even reflection about x- and y-axes. We do the same for $q_{2}^{s}\in \Omega$ defined by even reflections of $q_{2}^{*}$ about the square diagonals, and odd reflection about x- and y-axes and lastly, for the function $q_{3}^{s}\in \Omega$ defined by even reflections of $q_{3}^{*}$ about the square diagonals and x- and y-axes. By repeating arguments in [20], the new triple, $\left(q_{1}^{s},q_{2}^{s},q_{3}^{s}\right)$, is a weak solution of the Euler–Lagrange equations on Ω. One can verify that $\left(q_{1}^{s},q_{2}^{s},q_{3}^{s}\right)$ is a critical point of J on $A_{0}$ with the desired properties.

**Proposition 3.3.**

*For* A<0 *and* $L_{2}\neq 0$, *the critical point constructed in proposition 3.2, denoted by* $\left(q_{1}^{s},q_{2}^{s},q_{3}^{s}\right)$, *has non-constant* $q_{2}^{s}$ *on* Ω, *for all* λ>0.

**Proof.**

We proceed by contradiction. Assume that $q_{2}^{s}$ is constant on Ω. Recalling the boundary conditions (2.14), we necessarily have that $q_{2}^{s}\equiv 0$ in Ω. Substituting $q_{2}^{s}\equiv 0$ into (3.4), we obtain

3.15 $$q_{3}^{s}(x,y)=F(x)+G(y),$$

for arbitrary real-valued functions F,G, with $q_{3}^{s}=-s_{+}/6$ on ∂Ω. Therefore, $q_{3}^{s}\equiv -s_{+}/6$ in Ω. Substituting $q_{2}^{s}\equiv 0$ and $q_{3}^{s}\equiv -s_{+}/6$ into (3.3) and (3.5) yields

3.16 $$q_{1,yy}^{s}+q_{1,xx}^{s}=f(q_{1}^{s})$$

and

3.17 $$q_{1,yy}^{s}-q_{1,xx}^{s}=g(q_{1}^{s})+C_{g},$$

where

3.18 $$\begin{array}{rl} & f(q_{1}^{s})=\frac{4C\lambda^{2}}{(2+L_{2})L}{\left(q_{1}^{s}\right)}^{3}+\frac{2\lambda^{2}}{(2+L_{2})L}\left(A-\frac{Bs_{+}}{3}+\frac{Cs_{+}^{2}}{6}\right)q_{1}^{s}, \end{array}$$

3.19 $$\begin{array}{rl} & g(q_{1}^{s})=\frac{2\lambda^{2}}{LL_{2}}(B-Cs_{+}){\left(q_{1}^{s}\right)}^{2}, \end{array}$$

3.20 $$\begin{array}{rl} \mathrm{and}\; & C_{g}=-\frac{\lambda^{2}s_{+}}{LL_{2}}\left(A+\frac{Bs_{+}}{6}+\frac{Cs_{+}^{2}}{6}\right). \end{array}$$

From the reduced PDEs for $q_{1}^{s}$, (3.16) and (3.17), one can calculate

3.21 $$\begin{array}{rl} 2{\left(q_{1,xx}^{s}\right)}_{yy}-2{\left(q_{1,yy}^{s}\right)}_{xx} & \;=f^{\prime \prime }(q_{1}^{s})({\left(q_{1,y}^{s}\right)}^{2}-{\left(q_{1,x}^{s}\right)}^{2})-g^{\prime \prime }(q_{1}^{s})({\left(q_{1,y}^{s}\right)}^{2}+{\left(q_{1,x}^{s}\right)}^{2})\\ & \;\;+f^{\prime }(q_{1}^{s})(g(q_{1}^{s})+C_{g})-g^{\prime }(q_{1}^{s})f(q_{1}^{s}). \end{array}$$

Furthermore, from the symmetry properties of the constructed solution $q_{1}^{s}$ in proposition 3.2, we have

3.22 $$q_{1}^{s}{|}_{\left(0,0\right)}=q_{1,x}^{s}{|}_{\left(0,0\right)}=q_{1,y}^{s}{|}_{\left(0,0\right)}=0.$$

Substituting (3.22) into (3.21), we obtain

3.23a $$\begin{array}{rl} & \;(2q_{1,xxyy}^{s}-2q_{1,yyxx}^{s}){|}_{\left(0,0\right)}=(f^{\prime }(q_{1}^{s})C_{g}){|}_{\left(0,0\right)} \end{array}$$

3.23b $$\begin{array}{rl} & \;=-\frac{2\lambda^{2}}{(2+L_{2})L}\left(A-\frac{Bs_{+}}{3}+\frac{Cs_{+}^{2}}{6}\right)\frac{\lambda^{2}s_{+}}{LL_{2}}\left(A+\frac{Bs_{+}}{6}+\frac{Cs_{+}^{2}}{6}\right). \end{array}$$

If $A\neq -B^{2}/3C$, then equation (3.23*b*) at (0,0) is non-zero, which leads to a contradiction. If $A=-B^{2}/3C$, then $q_{3}^{s}\equiv -s_{+}/6=-B/6C$ and (3.17) reduces to $q_{1,yy}^{s}-q_{1,xx}^{s}=0,$ and hence, $q_{1}^{s}(x,y)=F_{1}(x-y)+F_{2}(x+y),$ for arbitrary real-valued functions $F_{1},F_{2}$.

From proposition (3.2), we know that for any λ>0, $q_{1}^{s}$ satisfies the symmetry property $q_{1}^{s}(x,y)=q_{1}^{s}(x,-y)$ and hence,

3.24 $$F_{1}(x-y)+F_{2}(x+y)=F_{1}(x+y)+F_{2}(x-y),\;(x,y)\in \Omega .$$

Subtracting $F_{2}(x-y)+F_{2}(x+y)$ on both sides of the equality (3.24), we get

3.25 $$G(z)=F_{1}(z)-F_{2}(z)\equiv K,\;z\in (-2,2),$$

for some constant K. The function $q_{1}^{s}$ may now be rewritten as

3.26 $$q_{1}^{s}(x,y)=F_{1}(x+y)+F_{1}(x-y)-K,\;(x,y)\in \Omega .$$

This formulation cannot be extended continuously on the boundary since, for (x,y)=(0,1), (−1,0) and (1,0), we have

3.27 $$F_{1}(1)+F_{1}(-1)-K=\frac{s_{+}}{2},\;2F_{1}(-1)-K=-\frac{s_{+}}{2},\;2F_{1}(1)-K=-\frac{s_{+}}{2},$$

which again leads to the required contradiction.

**Proposition 3.4.**

*There exists a critical edge length* $\lambda_{0}>0$ *such that, for any* $\lambda <\lambda_{0}$, *the critical point*, $\left(q_{1},q_{2},q_{3}\right)$, *in proposition 3.2 is the unique critical point of the LdG energy* (*3.6*).

**Proof.**

We adapt the uniqueness argument in lemma 8.2 of [21]. Let $\left(q_{1}^{\lambda },q_{2}^{\lambda },q_{3}^{\lambda }\right)$ be a global minimizer of J in (3.6), for λ>0. Let $(q_{1}^{\infty }(x),q_{2}^{\infty }(x),q_{3}^{\infty }(x))\in A_{0}$ be such that

3.28 $$f_{b}(q_{1}^{\infty }(\text{x}),q_{2}^{\infty }(\text{x}),q_{3}^{\infty }(\text{x}))=\min f_{b}=\frac{A}{3}s_{+}^{2}-\frac{2B}{27}s_{+}^{3}+\frac{C}{9}s_{+}^{4},$$

a.e. x∈Ω. Defining ${\bar{f}}_{b}(q_{1},q_{2},q_{3})=(1/L)(f_{b}(q_{1},q_{2},q_{3})-\min f_{b}(q_{1},q_{2},q_{3}))$, where L is constant, we have

3.29a $$\begin{array}{rl} & \;\int_{\Omega }f_{\mathrm{el}}(q_{1}^{\lambda },q_{2}^{\lambda },q_{3}^{\lambda })\;\text{dA}\leq \int_{\Omega }f_{\mathrm{el}}(q_{1}^{\lambda },q_{2}^{\lambda },q_{3}^{\lambda })+\lambda^{2}{\bar{f}}_{b}(q_{1}^{\lambda },q_{2}^{\lambda },q_{3}^{\lambda })\;\text{dA}\leq \int_{\Omega }f_{\mathrm{el}}(q_{1}^{\infty },q_{2}^{\infty },q_{3}^{\infty })\;\text{dA} \end{array}$$

3.29b $$\begin{array}{rl} & \;=:M_{2}(A,B,C,L_{2}), \end{array}$$

for some constant, $M_{2}>0$, depending only on A,B,C and $L_{2}$. Thus, we restrict ourselves to the following admissible space of Q-tensors:

3.30 $$A_{\mathrm{upper}}=\left\{\text{Q}:\int_{\Omega }|\nabla \text{Q}{|}^{2}\text{dA}\leq M_{2}(A,B,C,L_{2})\right\}.$$

The second derivatives of $f_{b}$ are quadratic polynomials in $\left(q_{1},q_{2},q_{3}\right)$. By an application of the relevant embedding theorem in [22] (theorem 9.16 which implies that for a bounded domain $\Omega \subset R^{N}$ with Lipschitz boundary, for any $u\in C_{c}^{1}(\Omega )$, $||u|{|}_{L^{p}}\leq c||u|{|}_{W^{1,2}},\;\forall p\in [N,\infty )$, with constant c depending only on Ω), there exist some constant $c_{0}$, depending only on A,B,C and Ω, such that

3.31 $${\left(\int_{\Omega }|f_{b}^{\prime \prime }{|}^{2}\text{dA}\right)}^{1/2}\leq c_{0}(A,B,C,\Omega ){\left(\int_{\Omega }|\nabla \text{Q}{|}^{2}\;\mathrm{dA}\right)}^{1/2}\leq c_{0}\sqrt{M_{2}}.$$

We apply the Hölder inequality to get, for any $x,y\in A_{\text{upper}}\cap A_{0}$,

3.32 $$\begin{array}{rl} & \;\int_{\Omega }f_{b}\left(\frac{x+y}{2}\right)-\frac{1}{2}f_{b}(x)-\frac{1}{2}f_{b}(y)\;\text{dA}\\ & \;\leq \frac{1}{8}\underset{A_{\mathrm{upper}}}{\sup}{\left(\int_{\Omega }|f_{b}^{\prime \prime }{|}^{2}\text{dA}\right)}^{1/2}{\left(\int_{\Omega }|x-y{|}^{4}\;\text{dA}\right)}^{1/2}\\ & \;\leq \frac{c_{0}\sqrt{M_{2}}}{8}||x-y|{|}_{L_{4}}^{2}. \end{array}$$

Therefore, for any $(q_{1},q_{2},q_{3}),(\tilde{q_{1}},\tilde{q_{2}},\tilde{q_{3}})\in A_{upper}\cap A_{0}$, we have

3.33a $$\begin{array}{rl} & \;\int_{\Omega }f_{b}\left(\frac{q_{1}+\tilde{q_{1}}}{2},\frac{q_{2}+\tilde{q_{2}}}{2},\frac{q_{3}+\tilde{q_{3}}}{2}\right)-\frac{1}{2}f_{b}(q_{1},q_{2},q_{3})-\frac{1}{2}f_{b}(\tilde{q_{1}},\tilde{q_{2}},\tilde{q_{3}})\;\mathrm{dA} \end{array}$$

3.33b $$\begin{array}{rl} & \;\leq c_{1}||q_{1}-\tilde{q_{1}},q_{2}-\tilde{q_{2}},q_{3}-\tilde{q_{3}}|{|}_{L_{4}}^{2} \end{array}$$

where $c_{1}=c_{1}(A,B,C,L_{2},\Omega )>0$. Using (3.11), an application of the Poincaré inequality, and repeating the same arguments as above, we have

3.34a $$\begin{array}{rl} & \;\int_{\Omega }f_{\mathrm{el}}(q_{1}-\tilde{q_{1}},q_{2}-\tilde{q_{2}},q_{3}-\tilde{q_{3}})\;\mathrm{dA} \end{array}$$

3.34b $$\begin{array}{rl} & \;\geq \min\{1,1+L_{2}\}\int_{\Omega }|\nabla (q_{1}-{\tilde{q}}_{1}){|}^{2}+|\nabla (q_{2}-{\tilde{q}}_{2}){|}^{2}+3|\nabla (q_{3}-\tilde{q_{3}}){|}^{2}\;\mathrm{dA}\\ & \;\geq \min\{1,1+L_{2}\}K(\Omega )(||q_{1}-{\tilde{q}}_{1}|{|}_{W^{1,2}}^{2}+||q_{2}-{\tilde{q}}_{2}|{|}_{W^{1,2}}^{2}+3||q_{3}-\tilde{q_{3}}|{|}_{W^{1,2}}^{2})\\ & \;\geq c_{2}(\Omega ,L_{2})||q_{1}-\tilde{q_{1}},q_{2}-\tilde{q_{2}},q_{3}-\tilde{q_{3}}|{|}_{L^{4}}^{2} \end{array}$$

for some constant, $c_{2}>0$, depending only on Ω and the sign of $L_{2}$. Using both (3.33*a*,b) and (3.34a,b), we have

3.35a $$\begin{array}{rl} & J\left[\frac{q_{1}+\tilde{q_{1}}}{2},\frac{q_{2}+\tilde{q_{2}}}{2},\frac{q_{3}+\tilde{q_{3}}}{2}\right] \end{array}$$

3.35b $$\begin{array}{rl} & \;\leq \frac{1}{2}J[q_{1},q_{2},q_{3}]+\frac{1}{2}J[\tilde{q_{1}},\tilde{q_{2}},\tilde{q_{3}}]-\frac{c_{2}}{8}||q_{1}-\tilde{q_{1}},q_{2}-\tilde{q_{2}},q_{3}-\tilde{q_{3}}|{|}_{L^{4}}^{2}\\ & \;\;-c_{1}\left(\frac{c_{2}}{8c_{1}}-\frac{\lambda^{2}}{L}\right)||q_{1}-\tilde{q_{1}},q_{2}-\tilde{q_{2}},q_{3}-\tilde{q_{3}}|{|}_{L^{4}}^{2},\\ & \;\leq \frac{1}{2}J[q_{1},q_{2},q_{3}]+\frac{1}{2}J[\tilde{q_{1}},\tilde{q_{2}},\tilde{q_{3}}] \end{array}$$

for $\lambda \leq \lambda_{0}:=\sqrt{c_{2}L/(8c_{1})}$. Thus, J is strictly convex for the finite energy triplets $\left(q_{1},q_{2},q_{3}\right)$, and has a unique critical point for $\lambda <\lambda_{0}$. Hence, the critical point constructed in proposition 3.2 is the unique minimizer of $J[q_{1},q_{2},q_{3}]$ and, in fact, the unique global LdG energy minimizer (when we consider Q-tensors with the full 5 d.f.), for sufficiently small λ.

**Lemma 3.5.**

*Let* $\left(q_{1},q_{2},q_{3}\right)$ *be the unique global minimizer of the energy* (3.6), *for* $\lambda <\lambda_{0}$ *given by proposition 3.4. Then for any* $L_{2}>-1$, *the function* $q_{1}:\Omega \to R$ *vanishes along the square diagonals* y=x *and* y=−x, *and the function* $q_{2}:\Omega \to R$ *vanishes along* y=0 *and* x=0.

**Proof.**

This is an immediate consequence of proposition 3.2, but we present an alternative short proof based on symmetry. Suppose that $\left(q_{1},q_{2},q_{3})\in W^{1,2}(\Omega ,R^{3}\right)$ is a global minimizer of J, in $A_{0}$ for a given λ>0. Then $\left(q_{1}(x,y),q_{2}(x,y),q_{3}(x,y)\right)$ is a solution of the Euler–Lagrange system (3.3)–(3.5), subject to the boundary conditions (2.13) and (2.14). So are the triples

$$(q_{1}(-x,y),-q_{2}(-x,y),q_{3}(-x,y)),\;(q_{1}(x,-y),-q_{2}(x,-y),q_{3}(x,-y)),\;(-q_{1}(y,x),q_{2}(y,x),q_{3}(y,x)),$$

that are compatible with the imposed boundary conditions. We combine this symmetry result with the uniqueness result in proposition 3.4 to get the desired conclusion. For example, use $q_{1}(x,y)=-q_{1}(y,x)$ with x=y to deduce that $q_{1}(x,x)=0$. Also, $q_{1}(-x,y)=q_{1}(x,y)$ with x=y yields that $q_{1}(x,-x)=q_{1}(x,x)=0$. Furthermore, we use the relation $q_{2}(x,y)=-q_{2}(-x,y)$ with x=0 to deduce $q_{2}(0,y)=0$, and similarly, $q_{2}(x,y)=-q_{2}(x,-y)$ with y=0 to obtain $q_{2}(x,0)=0$.

As in [8,15], we refer to the following dimensionless parameter in our numerical simulations:

$${\bar{\lambda }}^{2}:=\frac{2C\lambda^{2}}{L}.$$

**Figure 2.**
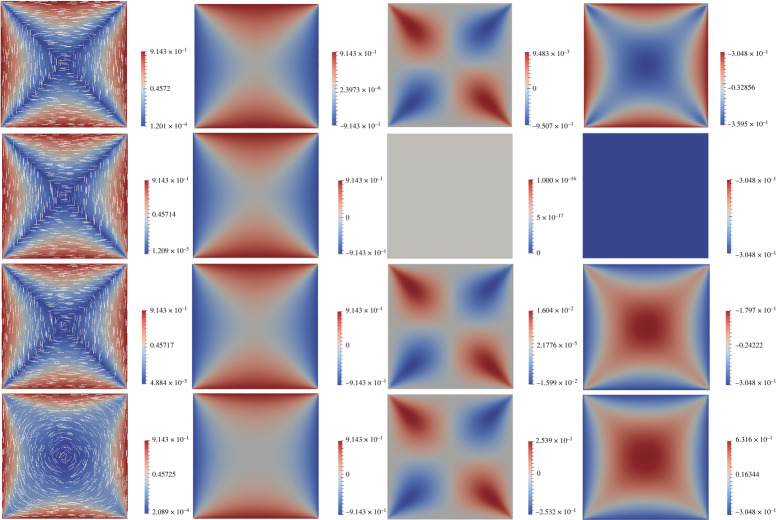
The unique stable solution of the Euler–Lagrange equations (3.3)–(3.5), with ${\bar{\lambda }}^{2}=5$, and (from the first to fourth row) $L_{2}=-0.5$, 0, 1 and 10, respectively. In the first column, we plot the scalar order parameter $s^{2}=q_{1}^{2}+q_{2}^{2}$ by colour from blue to red, and the director profile $n=(\cos(\arctan(q_{2}/q_{1})/2),\sin(\arctan(q_{2}/q_{1})/2))$ in terms of white lines. The $q_{1},q_{2}$ and $q_{3}$ profiles are plotted in the second to fourth columns, respectively. The same convention is used throughout the paper. (Online version in colour.)

**Figure 3.**
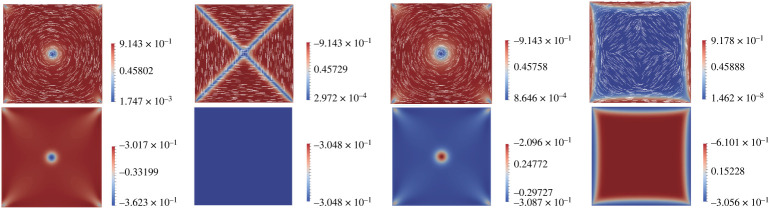
A solution branch for the system (3.3)–(3.5) with ${\bar{\lambda }}^{2}=500$, and $L_{2}=-0.5$, 0, 1 and 10 from left to right. This solution branch is a symmetric solution branch, as described in proposition 3.2, is unstable for $L_{2}=-0.5$, 0 and 1, and stable for $L_{2}=10$. We plot $s^{2}$ and n in the first row. and $q_{3}$ in the second row. (Online version in colour.)

In figure 2, we plot the unique stable solution of (3.3)–(3.5) with ${\bar{\lambda }}^{2}=5$, for $L_{2}=-0.5,0,1,10$. In this figure, and all subsequent figures, we fix $A=-B^{2}/3C$ with $B=0.64\times 10^{4}\;{\mathrm{Nm}}^{-2}$ and $C=0.35\times 10^{4}\;{\mathrm{Nm}}^{-2}$. When $L_{2}=0$, the solution is the WORS defined by (3.1). When $L_{2}=-0.5,1$, and 10, $q_{2}$ and $q_{3}$ are non-constant as proven above. One can check that $q_{1}:\Omega \to R$ vanishes along the square diagonals y=x and y=−x, and the function $q_{2}:\Omega \to R$ vanishes along y=0 and x=0, as proven in lemma 3.5. When $L_{2}=-0.5,1$ and 10, we observe a central +1-point defect in the profile of $\left(q_{1},q_{2}\right)$, and we label this as the $Ring^{+}$ solution. We then perform a parameter sweep of ${\bar{\lambda }}^{2}$, from 5 to 500, and find one of the symmetric solution branches in proposition 3.2, which is a continuation of the $Ring^{+}$ branch. The solutions with ${\bar{\lambda }}^{2}=500$ are plotted in figure 3. When $L_{2}=0$, we find the WORS for all λ>0. When $-1<L_{2}<0$, the solution exhibits a +1-defect at the square centre, continued from the $Ring^{+}$ branch and hence, we refer to it as the $Ring^{+}$ solution. When $L_{2}$ is positive and moderate in value, we again recover the $Ring^{+}$ solution branch and the corresponding $q_{3}<-s_{+}/6$ at the square centre for negative $L_{2}$, but $q_{3}>-s_{+}/6$ for positive $L_{2}$. When $L_{2}$ is large enough, we recover a symmetric solution which is approximately constant, $\left(0,0,s_{+}/3\right)$, away from the square edges, shown in the fourth column of figure 3 for $L_{2}=10$. We refer to this solution as the *Constant* solution throughout this manuscript.

## 4. Asymptotic studies

In the following, we work on a square domain without truncated vertices. For a truncated domain, we keep ε fixed, with short edges of length $\sqrt{2}\varepsilon$, where ε is sufficiently small.

### (a) The small λ and small anisotropy, $L_{2}\to 0$ limit

We work at the special temperature $A=-B^{2}/3C$ to facilitate comparison with the results in [23], where the authors investigate solution landscapes with $L_{2}=0$. Notably, for $L_{2}=0$ and $A=-B^{2}/3C$, reduced LdG solutions have $q_{3}\equiv -s_{+}/6=-B/6C$ for our choice of TBCs on 2D polygons, and it is natural to investigate the effects of the anisotropy parameter, $L_{2}$, in these 2D frameworks. At $A=-B^{2}/3C$, the governing Euler–Lagrange equations are given by the following system of partial differential equations:

4.1 $$\begin{array}{rl} & \left(1+\frac{L_{2}}{2}\right)\Delta q_{1}+\frac{L_{2}}{2}(q_{3,yy}-q_{3,xx})=\frac{\lambda^{2}}{L}q_{1}\left(-\frac{B^{2}}{3C}+2Bq_{3}+2C(q_{1}^{2}+q_{2}^{2}+3q_{3}^{2})\right),\\ & \left(1+\frac{L_{2}}{2}\right)\Delta q_{2}-L_{2}q_{3,xy}=\frac{\lambda^{2}}{L}q_{2}\left(-\frac{B^{2}}{3C}+2Bq_{3}+2C(q_{1}^{2}+q_{2}^{2}+3q_{3}^{2})\right), \end{array}$$

4.3 $$\begin{array}{rl} \mathrm{and}\; & \left(1+\frac{L_{2}}{6}\right)\Delta q_{3}+\frac{L_{2}}{6}(q_{1,yy}-q_{1,xx})-\frac{L_{2}}{3}q_{2,xy}\\ & \;\;=\frac{\lambda^{2}}{L}q_{3}\left(-\frac{B^{2}}{3C}-Bq_{3}+2C(q_{1}^{2}+q_{2}^{2}+3q_{3}^{2})\right)+\frac{\lambda^{2}B}{3L}(q_{1}^{2}+q_{2}^{2}), \end{array}$$

satisfying $q_{1}=q_{b}$, $q_{2}=0$ and $q_{3}=-B/6C$ on ∂Ω. We take a regular perturbation expansion of these functions in the $L_{2}\to 0$ limit. The leading-order approximation is given by the WORS, (q,0,−B/6C), where q is a solution of the Allen–Cahn equation, as in [7]:

4.4 $$\Delta q=\frac{2C\lambda^{2}}{L}q\left(q^{2}-\frac{B^{2}}{4C^{2}}\right),\;q=q_{b}\;\text{on}\;\partial \Omega .$$

We may assume that $q_{1},q_{2},q_{3}$ can be expanded in powers of $L_{2}$ as follows:

4.5 $$\begin{array}{rl} & q_{1}(x,y)=q(x,y)+L_{2}f(x,y)+\cdots \\ & q_{2}(x,y)=L_{2}g(x,y)+\cdots \\ \mathrm{and}\; & q_{3}(x,y)=-\frac{B}{6C}+L_{2}h(x,y)+\cdots \end{array}\}$$

for some functions f,g,h which vanish on the boundary. For λ small enough, one can show that there exists a unique solution $\left(f,g,h)\in W_{0}^{1,2}(\Omega ;R^{3}\right)$ with g≡0 and the symmetry property (−q(y,x),−f(y,x),g(y,x),h(y,x))=(q(−x,y),f(−x,y),g(−x,y),h(−x,y)), i.e. f(x,y)=0 on diagonals. Hence, for λ small enough, the cross structure of the WORS is lost mainly because of effects of $L_{2}$ on the component $q_{3}$, as we discuss below.

From [24], the solutions of (4.3) with λ=0, are a good approximation to the solutions of (4.3) for sufficiently small λ. When λ=0, $q=q_{0}$ where

4.6 $$\Delta q_{0}=0,\;(x,y)\in \Omega ,\;q_{0}=q_{1b},\;\text{on}\;\partial \Omega .$$

The analytical solution of (4.5) is given by [25]:

4.7 $$\begin{array}{rl} q_{0}(x,y) & \;=\frac{s_{+}}{2}\sum_{k\;odd}\frac{4}{k\pi }(\sin\left(\frac{k\pi (x+1)}{2}\right)\frac{\sinh(k\pi (1-y)/2)+\sinh(k\pi (1+y)/2)}{\sinh(k\pi )}\\ & \;-\sin\left(\frac{k\pi (y+1)}{2}\right)\frac{\sinh(k\pi (1-x)/2)+\sinh(k\pi (1+x)/2)}{\sinh(k\pi )}). \end{array}$$

The formula will also hold on a truncated square, with Dirichlet conditions on the truncated edges extracted from the explicit formula for $q_{0}$, i.e. we can choose boundary conditions on the short truncated edges that are compatible with $q_{0}$, once we compute $q_{0}$ on the full square domain without truncations. When λ=0, the unique solution of f,g,h in (4.4) is $f=f_{0}\equiv 0$, $g=g_{0}\equiv 0$ and $h=h_{0}$ where

**Figure 4.**
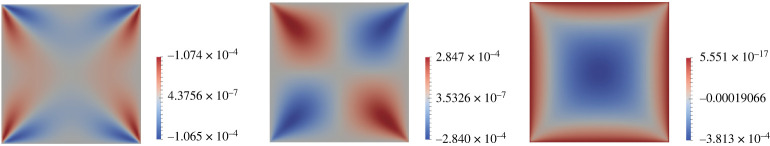
The difference between the solution of (3.3)–(3.5) with ${\bar{\lambda }}^{2}=0.01$, $L_{2}=0.1$ and asymptotic solution $\left(q_{0}+L_{2}f_{0},L_{2}g_{0},-(s_{+}/6)+L_{2}h_{0})=(q_{0},0,-(s_{+}/6)+L_{2}h_{0}\right)$. (Online version in colour.)

4.8 $$\Delta h_{0}=-\frac{1}{6}(q_{0,yy}-q_{0,xx}),$$

with $h_{0}=0$ on ∂Ω. See figure 4 for a numerical comparison between the asymptotic solution and relevant solutions of the Euler–Lagrange equations. The asymptotic solution in (4.3) is a good approximation of the solution of the Euler–Lagrange equations in (3.3)–(3.5) when ${\bar{\lambda }}^{2}$ is small enough (see figure 4).

**Proposition 4.1.**

*(Proof in electronic supplementary material) The analytical solution of* (4.7) *is given by*

4.9 $$h_{0}(x,y)=\sum_{m,n\;odd}\frac{16s_{+}mn}{3\pi^{2}{\left(m^{2}+n^{2}\right)}^{2}}\sin\left(\frac{m\pi (x+1)}{2}\right)\sin\left(\frac{n\pi (y+1)}{2}\right),$$

*where* $h_{0}(0,0)$ *is positive*.

### (b) The $L_{2}\to +\infty$ limit

Consider a regular perturbation expansion, in powers of $1/L_{2}$, of the solutions, $\left(q_{1},q_{2},q_{3}\right)$, of the Euler–Lagrange system (3.3)–(3.5), subject to (2.13) and (2.14). Let ρ,σ,τ be the leading-order approximations of $q_{1},q_{2},q_{3}$, respectively, in the $L_{2}\to \infty$ limit. Then we have:

4.10 $$\begin{array}{rl} & \frac{1}{2}\Delta \rho +\frac{1}{2}(\tau_{yy}-\tau_{xx})=0, \end{array}$$

4.11 $$\begin{array}{rl} & \frac{1}{2}\Delta \sigma -\tau_{xy}=0 \end{array}$$

4.12 $$\begin{array}{rl} \mathrm{and}\; & \frac{1}{6}\Delta \tau +\frac{1}{6}(\rho_{yy}-\rho_{xx})-\frac{1}{3}\sigma_{xy}=0. \end{array}$$

**Proposition 4.2.**

*The leading-order system in the* $L_{2}\to \infty$ *limit, given by* (4.9)–(4.11), *is not an elliptic PDE system*.

**Proof.**

The system of equations (4.9)–(4.11) can be written as

$$A{\text{q}}_{0,xx}+2B{\text{q}}_{0,xy}+C{\text{q}}_{0,yy}=\text{0},$$

where $q_{0}=(\rho ,\sigma ,\tau )$ and

$$A=\left(\begin{array}{ccc} \frac{1}{2} & 0 & -\frac{1}{2}\\ 0 & \frac{1}{2} & 0\\ -\frac{1}{6} & 0 & \frac{1}{6} \end{array}\right),\;B=\left(\begin{array}{ccc} 0 & 0 & 0\\ 0 & 0 & -\frac{1}{2}\\ 0 & -\frac{1}{6} & 0 \end{array}\right),\;C=\left(\begin{array}{ccc} \frac{1}{2} & 0 & \frac{1}{2}\\ 0 & \frac{1}{2} & 0\\ \frac{1}{6} & 0 & \frac{1}{6} \end{array}\right).$$

The system is said to be *elliptic*, in the sense of I.G. Petrovsky [26], if the determinant

$$|A\alpha^{2}+2B\alpha \beta +C\beta^{2}|\neq 0,$$

for any real numbers α,β≠0. We can check that

$$|A\alpha^{2}+2B\alpha \beta +C\beta^{2}|\equiv 0.$$

for any real numbers α,β. Hence, the limiting problem (4.9)–(4.11) is not an elliptic problem.

**Proposition 4.3.**

*There is no classical solution of the limiting problem* (4.9)–(4.11), *with the boundary conditions* (2.13) (*in the* ε→0 *limit*) *and* (2.14), *where* ε *is the short edge length of the truncated square*.

**Proof.**

As $L_{2}\to \infty$, the minimizers $\left(q_{1},q_{2},q_{3}\right)$ of J in (3.6), with $f_{\text{el}}$ as in (3.7), are constrained to satisfy

$$f_{\mathrm{div}}(q_{1},q_{2},q_{3})={\left(q_{1,x}+q_{2,y}-q_{3,x}\right)}^{2}+{\left(q_{2,x}-q_{1,y}-q_{3,y}\right)}^{2}=0,\;\text{a.e.}\;(x,y)\in \Omega ,$$

subject to the Dirichlet TBCs (2.13) and (2.14). Up to $O(L_{2})$, this corresponds to the following PDEs for the leading-order approximations ρ,σ,τ:

4.13 $${\left(\rho -\tau \right)}_{x}+\sigma_{y}=0$$

and

4.14 $$\sigma_{x}-{\left(\rho +\tau \right)}_{y}=0,$$

almost everywhere, subject to the same TBCs, $\rho =q_{b}$, σ=0, $\tau =-s_{+}/6$ on ∂Ω. As ε→0, the boundary conditions for ρ,σ,τ are piecewise constant, and hence the tangential derivatives of ρ,σ and τ vanish on the long square edges. On y=±1, the tangential derivative ${\left(\rho -\tau \right)}_{x}=0$, hence we obtain $\sigma_{y}=0$ in (4.12). Similarly, we have $\sigma_{x}=0$ on x=±1. This implies that $\partial_{\nu }\sigma =0$ on ∂Ω, where $\partial_{\nu }$ is the outward pointing normal derivative, and we view equation (4.10) to be of the form

$$\Delta \sigma =u(x,y),\;\partial_{\nu }\sigma {|}_{\partial \Omega }=0.$$

By the Hopf lemma, when $\partial_{\nu }\sigma =0$ on the boundary, we have σ≡0. Following the same arguments as in proposition 3.3, this requires that $\tau \equiv -s_{+}/6$. Substituting $\tau \equiv -s_{+}/6$ into equations (4.12) and (4.13), we obtain $\rho_{x}=\rho_{y}=0$, contradicting the boundary condition (2.13). Hence, there are no classical solutions of the system (4.9)–(4.11).

**Figure 5.**
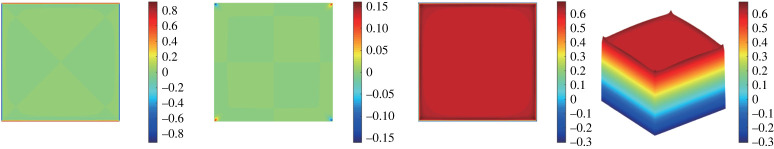
Solutions, ρ,σ,τ, (x,y,τ) of the leading-order system (4.9)–(4.11) in the $L_{2}\to \infty$ limit. (Online version in colour.)

Although there is no classical solution of (4.9)–(4.11) subject to the imposed boundary conditions, we can use finite difference methods to calculate a numerical solution, see figure 5. We label this solution $\left(\rho ,\sigma ,\tau )\equiv (0,0,s_{+}/3\right)$ on Ω as the *Constant* solution, where ρ and τ are discontinuous on ∂Ω. We now give a heuristic argument to explain the emergence of the *Constant* solution, as $L_{2}\to \infty$. With constant (ρ,σ,τ) in the interior of Ω, we have $f_{\text{div}}=0$ in Ω up to $O(L_{2})$. The choice of constant value $\left(\rho ,\sigma ,\tau )=(0,0,,s_{+}/3\right)$ is determined by the boundary conditions to minimize the elastic energy $f_{\text{div}}$. Therefore, ρ=σ=0, $\tau =s_{+}/3$ is the unique stable solution of (4.9)–(4.10), except for zero measure sets and we label $\left(q_{1},q_{2},q_{3})=(0,0,s_{+}/3\right)$ as the physically relevant Constant solution in the $L_{2}\to \infty$ limit. This is consistent with the numerical results in figure 5.

### (c) The λ→∞ limit

The set of minimizers of, $f_{b}$, in the $\left(q_{1},q_{2},q_{3}\right)$-plane can be written as $S=S_{1}\cup S_{2}$, where

4.15 $$S_{1}=\left\{(q_{1},q_{2},q_{3}):q_{1}^{2}+q_{2}^{2}=\frac{s_{+}^{2}}{4},\;q_{3}=-\frac{s_{+}}{6}\right\},\;S_{2}=\left\{\left(0,0,\frac{s_{+}}{3}\right)\right\}.$$

The λ→∞ limit is equivalent to the vanishing elastic constant limit, and $f_{b}$ converges uniformly to its minimum value in this limit [27].

**Proposition 4.4.**

*Let* $\Omega \in R^{2}$ *be a simply connected bounded open set with smooth boundary. Let* $\left(q_{1}^{\lambda },q_{2}^{\lambda },q_{3}^{\lambda }\right)$ *be a global minimizer of* $J(q_{1},q_{2},q_{3})$ *in the admissible class* $A_{0}$ *in* (3.9), *when* $L_{2}>-1$. *Then there exists a sequence* $\lambda_{k}\to \infty$ *such that* $\left(q_{1}^{\lambda_{k}},q_{2}^{\lambda_{k}},q_{3}^{\lambda_{k}})\to (q_{1}^{\infty },q_{2}^{\infty },q_{3}^{\infty }\right)$ *strongly in* $W^{1,2}(\Omega ;R^{3})$ *where* $(q_{1}^{\infty },q_{2}^{\infty },q_{3}^{\infty })\in S$. *If* $(q_{1}^{\infty },q_{2}^{\infty },q_{3}^{\infty })\in S_{1}$, *i.e*.

4.16 $$q_{1}^{\infty }=\frac{s_{+}}{2}\cos(2\theta^{\infty }),\;q_{2}^{\infty }=\frac{s_{+}}{2}\sin(2\theta^{\infty }),\;q_{3}^{\infty }=-\frac{s_{+}}{6},$$

*then* $\theta^{\infty }$ *is a minimizer of*

4.17 $$\int_{\Omega }|\nabla \theta {|}^{2}\;dA,$$

*in the admissible class* $A_{\theta }=\{\theta \in W^{1,2}(\Omega );\theta =\theta_{b}\;on\;\partial \Omega \}$. *The boundary condition*, $\theta_{b}$, *is compatible with* $\left(q_{1},q_{2}\right)$ *on* ∂Ω *by the relation* $\left(q_{1b},q_{2b})=(s_{+}/2)(\cos(2\theta_{b}),\sin(2\theta_{b})\right)$. *Otherwise*, $(q_{1}^{\infty },q_{2}^{\infty },q_{3}^{\infty })(x,y)\in S_{2}$, *i.e.*

4.18 $$(q_{1}^{\infty },q_{2}^{\infty },q_{3}^{\infty })=\left(0,0,\frac{s_{+}}{3}\right).$$

**Proof.**

Our proof is analogous to lemma 3 of [27]. See electronic supplementary material.

This proposition cannot be applied to square domains directly, because $\theta_{b}$ cannot be defined at the square vertices. The TBCs necessarily mean that $\theta_{b}$ is constant on each square edge, and hence, discontinuous at the vertices. However, we can still use the Proposition above to understand the qualitative properties of energy minimizers in the λ→∞ limit, by smoothening the boundary near the vertices and by defining $\theta_{b}$ appropriately. Firstly, the TBCs imply that $\theta_{b}$ must be a multiple of π on the horizontal edges, and an odd multiple of π/2 on the vertical square edges. Secondly, we prescribe $\theta_{b}$ so that the degree of $n_{b}=(\cos\theta_{b},\sin\theta_{b})$ is zero on the square boundary. For example, experiments suggest that there are two classes of stable equilibria, which are almost in the set $S_{1}$, for large λ—the diagonal and rotated states. The diagonal states, D, are such that the nematic director (in the plane) is aligned along one of the square diagonals. The rotated states, labelled as R states, are such that the director rotates by π radians between a pair of opposite square edges. There are two rotationally equivalent D states, and four rotationally equivalent R states. The corresponding boundary conditions in terms of θ are given by $\theta_{b}=\theta_{b}^{D}$ or $\theta_{b}^{R}$, respectively, where

4.19 $$\left\{ \begin{array}{l} \theta_{b}^{D}=\frac{\pi }{2},\;\text{on}\;x=\pm 1,\\ \theta_{b}^{D}=0,\;\;\text{on}\;y=\pm 1, \end{array}\right.\;\left\{ \begin{array}{l} \theta_{b}^{R}=\frac{\pi }{2},\;\text{on}\;x=-1,\\ \theta_{b}^{R}=-\frac{\pi }{2},\;\text{on}\;x=1,\\ \theta_{b}^{R}=0,\;\;\;\text{on}\;y=\pm 1. \end{array}\right.$$

These conditions can be translated to Dirichlet conditions for $\theta_{b}$ on the truncated square as well; for example, we can solve Δθ=0 on a square domain, subject to these boundary conditions and use this solution to prescribe the Dirichlet conditions on the short edges of the truncated square.

**Figure 6.**
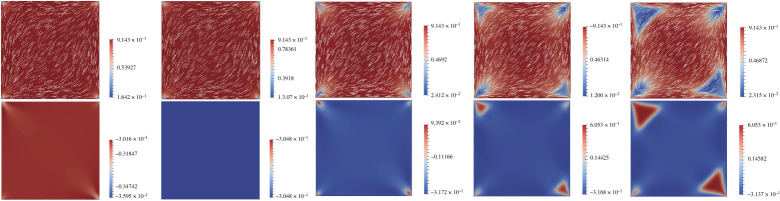
The D solution with ${\bar{\lambda }}^{2}=1000$, with $L_{2}=-0.5,0,10,30$ and 45, respectively. In the first row, we plot $s^{2}$ and n. In the second row, we plot the $q_{3}$-profiles. (Online version in colour.)

In figure 6, we study the effect of increasing $L_{2}$ on a D state with ${\bar{\lambda }}^{2}=1000$. When $L_{2}=0$, we see that $s^{2}=q_{1}^{2}+q_{2}^{2}\approx s_{+}^{2}/4$, $q_{3}=-s_{+}/6$ almost everywhere. In [13], the authors show that the limiting profiles described in proposition 4.4 are a good approximation to the solutions of (3.3)–(3.5), for large λ. The differences between the limiting profiles and the numerically computed D solutions concentrate around the vertices, for large λ. As $|L_{2}|$ increases, $q_{3}$ deviates significantly from the limiting value $q_{3}^{\infty }=-s_{+}/6$, near the square vertices. From an optical perspective, we expect to observe larger defects near the square vertices for more anisotropic materials with $L_{2}\gg 1$, on large square domains.

In [25], the authors compute the limiting energy, $J_{\infty }$, of D and R solutions on a unit square to be:

4.20 $$J_{\infty }(D)=2\pi s_{+}^{2}\left(1+\frac{L_{2}}{2}\right)\left(\ln\left(\frac{1}{\varepsilon }\right)+\ln\left(\frac{2}{\pi }\right)+s_{1}-s_{2}+O(\varepsilon^{2})\right)$$

and

4.21 $$J_{\infty }(R)=2\pi s_{+}^{2}\left(1+\frac{L_{2}}{2}\right)\left(\ln\left(\frac{1}{\varepsilon }\right)+\ln\left(\frac{2}{\pi }\right)+s_{1}+s_{2}+O(\varepsilon^{2})\right),$$

respectively, where

$$s_{1}=2\sum_{n=0}^{\infty }\frac{\coth((2n+1)\pi )-1}{2n+1}\;\mathrm{and}\;s_{2}=2\sum_{n=0}^{\infty }\frac{\mathrm{csch}((2n+1)\pi )}{2n+1}.$$

Since csch((2n+1)π) is positive, we have $J_{\infty }(D)<J_{\infty }(R)$. The numerical values of $\text{ln}(2/\pi )+s_{1}-s_{2}$ and $\text{ln}(2/\pi )+s_{1}+s_{2}$ are approximately zero, so $J_{\infty }(D)$ and $J_{\infty }(R)$ are approximately ln(1/ε) for small ε, and the limiting energies are linear in $L_{2}$.

The Constant solution $q_{c}\equiv (0,0,s_{+}/3)$ has transition layers on the boundary from $\left(0,0,s_{+}/3\right)$ to $\left(s_{+}/2,0,-s_{+}/6\right)$ or $\left(-s_{+}/2,0,-s_{+}/6\right)$. The limiting energy in (4.16) is the same for the $L_{2}=0$ and $L_{2}\neq 0$ cases. Therefore, there are no additional complexities from the $L_{2}$ term. Analogous to section 4 of [15], using classical arguments in the theory of Γ-convergence, the limiting energy of the Constant solution is the sum of four transition costs: $d((\pm s_{+}/2,0,-s_{+}/6),(0,0,s_{+}/3))$, where $d(q_{0}^{*},q_{1}^{*})$ is the geodesic distance between $q_{0}^{*}$ and $q_{1}^{*}$ associated with the Riemannian metric $F^{1/2}$, where $F=f_{b}-\min f_{b}$. The numerical value of $d((\pm s_{+}/2,0,-s_{+}/6),(0,0,s_{+}/3))$ is 41.6817 in [15]. The limiting energy $G_{\infty }(Constant)=4d((\pm s_{+}/2,0,-s_{+}/6),(0,0,s_{+}/3))$ is independent of $L_{2}$. Hence, there is a critical value

$$L_{2}^{*}=\frac{4c_{1}}{s_{+}^{2}\pi (\ln(1/\varepsilon )+\ln(2/\pi )+s_{1}-s_{2}+O(\varepsilon^{2}))}-2,$$

such that for $L_{2}>L_{2}^{*}$, the limiting Constant solution is energetically preferable to the D and R solutions, i.e. $G_{\infty }(\text{Constant})<J_{\infty }(D)<J_{\infty }(R)$.

### (d) The Novel *pWORS* solutions

For all λ>0 and $L_{2}=0$, the WORS is a solution of (3.3)–(3.5) given by (q,0,−B/6C), where q satisfies (4.3). In §4a, we study the Euler–Lagrange equations, in the small λ and small $L_{2}$ limit, up to $O(L_{2})$; see (4.4). However, g≡0 is a solution for all λ and we assume that the solution, $\left(q_{1},q_{2},q_{3}\right)$, of (3.3)–(3.5), can be expanded as follows:

4.22 $$\begin{array}{rl} & q_{1}(x,y)=q(x,y)+L_{2}f(x,y)+L_{2}^{2}\varphi (x,y)+\cdots , \end{array}$$

4.23 $$\begin{array}{rl} & q_{2}(x,y)=0+L_{2}g(x,y)+L_{2}^{2}\gamma (x,y)+\cdots \end{array}$$

4.24 $$\begin{array}{rl} \mathrm{and}\; & q_{3}(x,y)=-\frac{B}{6C}+L_{2}h(x,y)+L_{2}^{2}\mu (x,y)+\cdots \end{array}$$

**Figure 7.**
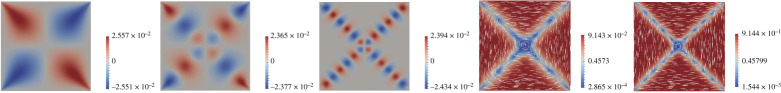
Left three figures: plots of γ in (4.22), with ${\bar{\lambda }}^{2}=5$, 100 and 500 from the left to right. Right two figures: the configurations of the numerically computed pWORS with $L_{2}=3.5$, ${\bar{\lambda }}^{2}=350$ and 1000. (Online version in colour.)

In figure 7, we plot a branch of the γ solutions. As λ increases, we observe an increasing number of zeroes on the square diagonals, where γ=0. For any λ>0, we can use the initial condition $\left(q_{1},q_{2},q_{3})=(q+L_{2}f,L_{2}g+L_{2}^{2}\gamma ,-(s_{+}/6)+L_{2}h\right)$ to numerically find a new branch of unstable solutions, referred to as pWORS configurations in figure 7. In the $\left(q_{1},q_{2}\right)$ plane, the pWORS has a constant set of eigenvectors away from the diagonals, and has multiple ±1/2-point defects on the two diagonals, so that the pWORS is similar to the WORS away from the square diagonals. As λ increases, the number of alternating +1/2 and −1/2 point defects on the square diagonals increases, for the numerically computed pWORS. This is mirrored by the function γ that encodes the second-order effect of $L_{2}$ on the WORS.

## 5. Bifurcation diagrams

We use the open-source package FEniCS [28] to perform all the finite-element simulations, numerical integration and stability checks in this paper [29,30]. We apply the finite-element method on a triangular mesh with mesh-size h≤1/256, for the discretization of a square domain. The non-linear equations (3.3)–(3.5) are solved by Newton’s methods with a linear LU solver at each iteration. The tolerance for convergence is set to $1\times 10^{-13}$. We check the stability of the numerical solution by numerically calculating the smallest real eigenvalue of the Hessian matrix of the energy functional (3.6), using the LOBPCG (locally optimal block preconditioned conjugate gradient) method [31]. If the smallest real eigenvalue is negative, the solution is unstable, and stable otherwise. In what follows, we compute bifurcation diagrams for the solution landscapes, as a function of ${\bar{\lambda }}^{2}$, with fixed temperature $A=-B^{2}/3C$, for five different values of $L_{2}=0,1,2.6,3,10$. The C and L are fixed material-dependent constants, so $\bar{\lambda }$ is proportional to λ and we will use these diagrams to infer qualitative solution trends. in terms of the edge length, λ.

For λ small enough, there is a unique solution for any value of $L_{2}$; see the results in §4. For $L_{2}=0$, the unique stable solution, for small enough λ, is the WORS. The unique solution deforms to the ${Ring}^{+}$, with a central point defect, for relatively small $L_{2}>0$ such as $L_{2}=1,2.6$. For $L_{2}=10$, the unique solution is the *Constant* solution, on the grounds that this solution approaches the constant state, $\left(q_{1},q_{2},q_{3})\to (0,0,s_{+}/3\right)$, in the square interior as λ→∞. The $Ring^{+}$ and Constant solution branches coexist for some values of $L_{2}$ ($2.7\leq L_{2}\leq 3.4$ for ${\bar{\lambda }}^{2}=100$, $2.85\leq L_{2}\leq 5.5$ for ${\bar{\lambda }}^{2}=200$). When $L_{2}$ is large enough, the Constant solution has lower energy than the $Ring^{+}$ solution.

**Figure 8.**
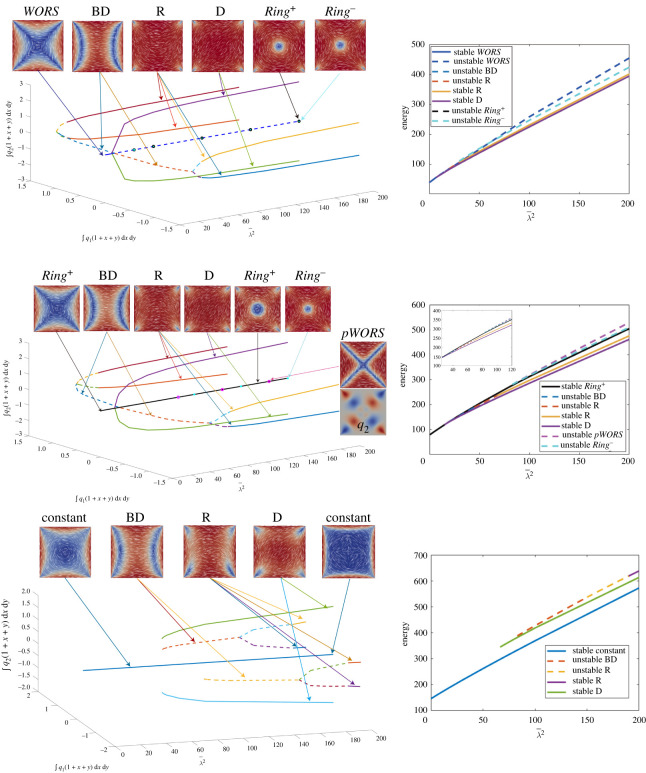
Bifurcation diagrams with $L_{2}=0$, 2.6 and 10 from top to bottom. Left: plot of $\int q_{1}(1+x+y)\;\text{d}x\;\text{d}y$, $\int q_{2}(1+x+y)\;\text{d}x\;\text{d}y$ verses ${\bar{\lambda }}^{2}$. Right: plot of the energy $J-\int_{\Omega }\min f_{b}\;\mathrm{dA}$ verses ${\bar{\lambda }}^{2}$. (Online version in colour.)

We distinguish between the distinct solution branches by defining two measures $\int_{\Omega }q_{1}(1+x+y)dxdy$ and $\int_{\Omega }q_{2}(1+x+y)\;dx\;dy$. In addition to the WORS, ${Ring}^{+}$, *Constant* solutions, there also exist the unstable ${Ring}^{-}$ and unstable pWORS solution branches with the same symmetries in proposition 3.2, which are indistinguishable by these measures. Hence, they appear on the same line in bifurcation diagram in figure 8 for all $L_{2}>0$. The difference between the ${Ring}^{+}$, ${Ring}^{-}$, WORS, Constant and pWORS can be spotted from the associated $q_{2}$-profiles. If $q_{2}<0$ on x=y and x>0, the corresponding solution is the ${Ring}^{+}$ solution. If $q_{2}>0$ on x=y and x>0, the corresponding solution is the $Ring^{-}$ solution. The ${Ring}^{+}$ and ${Ring}^{-}$ solutions also exist for $L_{2}=0$. If $q_{2}\equiv 0$, the solution is either the WORS or Constant solutions. If $q_{2}$ has isolated zero points on the square diagonals, the corresponding solution is identified to be the pWORS solution branch.

We numerically solve the Euler–Lagrange equations (3.3)–(3.5) with ${\bar{\lambda }}^{2}=0.1$ by using Newton’s method to obtain the unique stable solutions for the different values of $L_{2}$. The initial condition is not important here, since the solution is unique and the nonlinear term is small for ${\bar{\lambda }}^{2}=0.1$. We perform an increasing ${\bar{\lambda }}^{2}$ sweep for the WORS, ${Ring}^{+}$ and Constant solution branches and a decreasing ${\bar{\lambda }}^{2}$ sweep for the diagonal D, and rotated R solution branches (as described in §4c). The stable ${Ring}^{+}$ branch for $L_{2}=3$ is obtained by taking the stable ${Ring}^{+}$ branch, with $L_{2}=2.6$ as the initial condition. The unstable WORS and $Ring^{+}$ are tracked by continuing the stable WORS and stable $Ring^{+}$ branches. If the $Ring^{+}$ branch is given by $\left(q_{1},q_{2},q_{3}\right)$ for a fixed $L_{2}>0$, then the initial condition for the unstable $Ring^{-}$-solution is given by the corresponding $\left(q_{1},-q_{2},q_{3}\right)$ solution, for any λ>0. The initial condition for the unstable pWORS branch is given by $\left(q_{1},q_{2},q_{3})=(q+L_{2}f,L_{2}g+L_{2}^{2}\gamma ,-(s_{+}/6)+L_{2}h\right)$, where q,f,g,h,γ are $O(L_{2})$ perturbations in (4.4) and $O(L_{2}^{2})$ perturbation in (4.22), for any λ>0 (figure 7).

Consider the case $L_{2}=0$. For $\lambda <\lambda^{*}$, there is the unique WORS. For $\lambda =\lambda^{*}$, the stable WORS bifurcates into an unstable WORS, and two stable D solutions. When $\lambda =\lambda^{**}>\lambda^{*}$, the unstable WORS bifurcates into two unstable BD, which are featured by isotropic lines or defect lines localized near a pair of opposite square edges. When $\lambda =\lambda^{***}>\lambda^{**}$, unstable ${Ring}^{\pm }$ solutions appear simultaneously. When $L_{2}=0$, the ${Ring}^{+}$ and ${Ring}^{-}$ solution have the same energy. Each unstable BD further bifurcates into two unstable R solutions. As λ increases, the unstable R solutions gain stability. The WORS has the highest energy amongst the numerically computed solutions for $L_{2}=0$, for large λ. For $L_{2}\neq 0$, the WORS ceases to exist and the unique solution is the stable $Ring^{+}$ solution. For $L_{2}=2.6$, the ${Ring}^{+}$ solution is stable for ${\bar{\lambda }}^{2}\leq 200$ and the unstable pWORS and ${Ring}^{-}$ appear for large λ. At the first bifurcation point $\lambda =\lambda^{*}$, the $Ring^{+}$ bifurcates into two stable D solutions. At the second bifurcation point, $\lambda =\lambda^{**}>\lambda^{*}$, it further bifurcates into two unstable BD solutions and for $\lambda =\lambda^{***}>\lambda^{**}$, the unstable ${Ring}^{-}$ and unstable pWORS solution branches appear. The $Ring^{-}$ and pWORS are always unstable and the $Ring^{+}$ solution has slightly lower energy than the $Ring^{-}$. The unstable pWORS has higher energy than the unstable $Ring^{\pm }$ solutions when λ is large. For larger $L_{2}$, the unique stable solution, for small λ, is the Constant solution, which remains stable for ${\bar{\lambda }}^{2}\leq 200$. We can clearly see that the Constant solution approaches $\left(q_{1},q_{2},q_{3})\to (0,0,s_{+}/3\right)$ as λ gets large. For $L_{2}=10$, the pWORS and ${Ring}^{\pm }$ states disappear, and the Constant solution does not bifurcate to any known states. The BD and D solution branches are now disconnected from the stable Constant solution branch. As we perform a decreasing ${\bar{\lambda }}^{2}$ sweep for the D or BD solution branches, we cannot find a D or BD solution for $\lambda <\lambda^{D}$ or $\lambda <\lambda^{BD}$, for small $\lambda^{D}$ and $\lambda^{BD}$. The Constant solution has lower energy than the R and D solutions for large λ, as suggested by the estimates in §4c. For much larger values of $L_{2}$, we only numerically observe the Constant solution branch.

To summarize, the primary effect of the anisotropy parameter, $L_{2}$, is on the unique stable solution for small λ. The elastic anisotropy destroys the cross structure of the WORS, and also enhances the stability of the $Ring^{+}$ and Constant solutions. A further interesting feature for large $L_{2}$, is the disconnectedness of the D and R solution branches from the parent Constant solution branch. This indicates novel hidden solutions for large $L_{2}$, which may have different structural profiles to the discussed solution branches, and this will be investigated in greater detail, in future work.

In the next proposition (proof in electronic supplementary material), we prove a stability result which gives partial insight into the stabilizing effects of positive $L_{2}$. Let $\left(q_{1},q_{2},q_{3}\right)$ be an arbitrary critical point of the energy functional (3.6). As is standard in the calculus of variations, we say that a critical point is locally stable if the associated second variation of the energy (3.6) is positive for all admissible perturbations, and is unstable if there exists an admissible perturbation for which the second variation is negative.

**Proposition 5.1.**

*For* $L_{2}\geq (\lambda^{2}/L)c(A,B,C,\Omega )$, *where* c *is some constant depending only on* A,B,C *and* Ω, *the critical points of the energy functional* (*3.6*) *in the restricted admissible space*

$$\begin{array}{rl} A_{*} & \;=\{(q_{1},q_{2},q_{3})\in A_{0}:\int_{\Omega }|\nabla q_{1}{|}^{2}\leq M_{1}(A,B,C),\\ & \;\int_{\Omega }|\nabla q_{2}{|}^{2}\leq M_{2}(A,B,C),\int_{\Omega }|\nabla q_{3}{|}^{2}\leq M_{3}(A,B,C)\}, \end{array}$$

*are locally stable with respect to the perturbations*

5.1 $$\text{V}(x,y)=v_{1}(x,y)(\hat{\text{x}}⊗\hat{\text{x}}-\hat{\text{y}}⊗\hat{\text{y}})+v_{2}(x,y)(\hat{\text{x}}⊗\hat{\text{y}}+\hat{\text{y}}⊗\hat{\text{x}})$$

*and*

5.2 $$\text{V}(x,y)=v_{3}(x,y)(2\hat{\text{z}}⊗\hat{\text{z}}-\hat{\text{x}}⊗\hat{\text{x}}-\hat{\text{y}}⊗\hat{\text{y}}).$$

## 6. Conclusion and discussions

We study the effects of elastic anisotropy on stable nematic equilibria on a square domain, with TBCs, primarily focusing on the interplay between the square edge length, λ, and the elastic anisotropy, $L_{2}$. We study LdG critical points with three degrees of freedom, $q_{1},q_{2},q_{3}$. We use symmetry arguments on an 1/8th of the square domain, to construct symmetric LdG critical points for which $q_{1}$ vanishes on the square diagonals, and $q_{2}$ vanishes on the coordinate axes. The WORS is a special symmetric critical point for $L_{2}=0,$ with $q_{2}\equiv 0$ . In particular, $q_{2}$ cannot be identically zero for $L_{2}\neq 0$. There are different classes of these symmetric critical points, and we perform asymptotic studies in the small λ and small $L_{2}$ limit, and large $L_{2}$ limits, to provide good asymptotic approximations for the novel $Ring^{+}$ and Constant solutions, both of which can be stable in physically relevant regimes. The large λ-picture for $L_{2}\neq 0$ is qualitatively similar to the $L_{2}=0$ case, with the stable diagonal, D and rotated, R solutions. The notable difference is the emergence of the competing stable Constant solution, which is energetically preferable to the D and R-solutions, for large $L_{2}$ and large λ. This suggests that for highly anisotropic materials with large $L_{2}$, the experimentally observable state is the Constant solution with $q_{1}^{2}+q_{2}^{2}\approx 0$ in the square interior. In other words, the Constant state is almost perfectly uniaxial, with uniaxial symmetry along the z-direction, and will offer highly contrasting optical properties compared to the D and R solutions. This offers novel prospects for multistability for highly anisotropic materials.

Another noteworthy feature is the stabilizing effect of $L_{2}$, as discussed in §5. The ${Ring}^{+}$ solution has a central point defect in the square interior and is unstable for $L_{2}=0$. However, it gains stability for moderate values of λ, as $L_{2}$ increases, and ceases to exist for very large positive values of $L_{2}$. We note some similarity with recent work on ferronematics [32], where the coupling between the nematic director and an induced spontaneous magnetization stabilizes interior nematic point defects, with $L_{2}=0$. It remains an open question as to whether elastic anisotropy or coupling energies (perhaps with certain symmetry and invariance properties) can stabilize interior nematic defects for tailor-made applications.

## Data Availability

All data and materials relating to this research are presented in the manuscript. The data are provided in electronic supplementary material [33].
